# The role of dietary fatty acids in predicting myocardial structure in fat-fed rats

**DOI:** 10.1186/1476-511X-10-92

**Published:** 2011-06-07

**Authors:** Kimberly M Jeckel, Kelsey E Miller, Adam J Chicco, Phillip L Chapman, Christopher M Mulligan, Paul H Falcone, Melissa L Miller, Michael J Pagliassotti, Melinda A Frye

**Affiliations:** 1 Department of Biomedical Sciences, Campus delivery #1680, College of Veterinary Medicine and Biomedical Sciences, Colorado State University, Fort Collins, CO 80523, USA; 2 Department of Health and Exercise Science, Campus delivery #1582, College of Applied Human Sciences, Colorado State University, Fort Collins, CO 80523, USA; 3 Department of Statistics, Campus delivery #1877, College of Natural Sciences, Colorado State University, Fort Collins, CO 80523, USA; 4 Department of Food Science and Human Nutrition, Campus delivery #1571, College of Applied Human Sciences, Colorado State University, Fort Collins, CO 80523, USA

**Keywords:** Obesity, Cardiomyopathy, Polyunsaturated fatty acids, Left ventricular hypertrophy

## Abstract

***Background*:**

Obesity increases the risk for development of cardiomyopathy in the absence of hypertension, diabetes or myocardial ischemia. Not all obese individuals, however, progress to heart failure. Indeed, obesity may provide protection from cardiovascular mortality in some populations. The fatty acid milieu, modulated by diet, may modify obesity-induced myocardial structure and function, lending partial explanation for the array of cardiomyopathic phenotypy in obese individuals.

***Methods*:**

Adult male Sprague-Dawley rats were fed 1 of the following 4 diets for 32 weeks: control (CON); 50% saturated fat (SAT); 40% saturated fat + 10% linoleic acid (SAT+LA); 40% saturated fat + 10% α-linolenic acid (SAT+ALA). Serum leptin, insulin, glucose, free fatty acids and triglycerides were quantitated. *In vivo *cardiovascular outcomes included blood pressure, heart rate and echocardiographic measurements of structure and function. The rats were sacrificed and myocardium was processed for fatty acid analysis (TLC-GC), and evaluation of potential modifiers of myocardial structure including collagen (Masson's trichrome, hydroxyproline quantitation), lipid (Oil Red O, triglyceride quantitation) and myocyte cross sectional area.

***Results*:**

Rats fed SAT+LA and SAT+ALA diets had greater cranial LV wall thickness compared to rats fed CON and SAT diets, in the absence of hypertension or apparent insulin resistance. Treatment was not associated with changes in myocardial function. Myocardial collagen and triglycerides were similar among treatment groups; however, rats fed the high-fat diets, regardless of composition, demonstrated increased myocyte cross sectional area.

***Conclusions*:**

Under conditions of high-fat feeding, replacement of 10% saturated fat with either LA or ALA is associated with thickening of the cranial LV wall, but without concomitant functional changes. Increased myocyte size appears to be a more likely contributor to early LV thickening in response to high-fat feeding. These findings suggest that myocyte hypertrophy may be an early change leading to gross LV hypertrophy in the hearts of "healthy" obese rats, in the absence of hypertension, diabetes and myocardial ischemia.

## Background

In the United States the prevalence of overweight and obese adults averages 26% nationally,[1] having increased nearly 20% over the last 3 decades[2]. Beyond the human toll lies the economic cost that is projected to be 900 billion by the year 2030[3]. Obese individuals have a higher risk of morbidity and mortality attributed to cardiovascular disease,[4] and specifically are at higher risk for the development of cardiomyopathy leading to heart failure[5,6]. Obesity-mediated cardiomyopathy (OC) and heart failure have traditionally been attributed to hypertension, myocardial ischemia and diabetes. More recently, increased left ventricular (LV) mass and myocardial dysfunction have been associated with obesity in otherwise healthy humans (i.e. without concomitant hypertension, ischemic heart disease or apparent insulin resistance)[7-11]. Left ventricular hypertrophy (LVH) is an early echocardiographic change that reflects increased LV mass. This structural change is commonly identified in obese individuals,[12] and LV mass has been positively associated with adiposity and body mass index[11,13,14]. Importantly, LVH is an independent risk factor for development of systolic dysfunction,[15] and is associated with an increased risk for cardiovascular and all-cause mortality in people[16-18].

It is unknown why some obese individuals progress to heart failure, while others appear to be protected from mortality[19]. It is possible that diet composition is one factor that predicts the cardiac phenotype in response to obesity, and therefore disease progression. There is evidence that the fatty acid milieu predicts structural and functional changes in the heart that occur with obesity. Saturated and n-6 polyunsaturated fatty acids (PUFA) enhance myocyte apoptosis and necrosis,[20,21] while monounsaturated and n-3 PUFA attenuate apoptosis in cardiac and endothelial cells[22,23]. In addition, feeding of n-6 PUFA to normal pigs was associated with myocardial inflammation, while feeding

n-3 PUFA was associated with anti-inflammatory effects[24]. Further, dietary fat composition may differentially impact LV structure and contractile function,[25,26] and studies of cultured myocytes support this idea[27].

Collectively, these findings suggest that LVH may be an important early event in the development of myocardial dysfunction in obese individuals. At the cellular level, a thickened LV may be attributed to extracellular matrix (ECM) remodeling, myocardial lipid accumulation and/or cardiac myocyte hypertrophy. There is evidence that these processes are differentially expressed according to dietary fat, so were chosen for emphasis in the present study. The human population more frequently experiences obesity as a result of nutritional and lifestyle factors compared to genetic aberrancy; thus, a dietary obese model was chosen for this study. We propose that defining alterations in cardiac structure and function attributable to obesity may be best accomplished by investigating the effects of combined fatty acid moieties from a dietary source, in the *in vivo *setting of intact anti-inflammatory and antioxidant systems.

The purpose of this study was to determine whether the heterogeneous phenotype of OC might be partially attributed to dietary fatty acid composition. Primary outcomes included myocardial structure and function, measured echocardiographically, in addition to ECM remodeling, myocardial lipid accumulation and cardiac myocyte hypertrophy. To understand the morphologic and metabolic milieu within which primary outcomes were measured, we secondarily characterized adiposity, hemodynamics, serum metabolic indices and myocardial fatty acid composition.

We hypothesized that long-term feeding of a high saturated fat diet would be associated with LVH, and that concomitant intake of n-6 PUFA and n-3 PUFA would exacerbate and attenuate, respectively, this early structural change. Further, we hypothesized that despite the presence of LVH, myocardial function, measured echocardiographically, would remain intact in these dietary obese rats. Regarding contributors to LV thickening, it was anticipated that intake of a diet high in saturated fat and n-6 PUFA would result in the most profound lesion severity, compared with other high fat diets tested.

## Methods

### Animals

Adult male Sprague-Dawley (SD) rats (CD^® ^IGS Rat, Charles River Laboratories, Wilmington, MA) were maintained in the Colorado State University Laboratory Animal Resource Center in a temperature- and humidity-controlled environment. Rats were housed in pairs with a normal 12-hour light/12 hour dark cycle. Protocols and conditions within the facility meet or exceed the standards for animal housing facilities as described in the Animal Welfare Act regulations, the Guide for the Care and Use of Laboratory Animals and the Guide for the Care and Use of Agricultural Animals in Agricultural Research and Teaching. The rats were allowed a 2-week acclimation period prior to initiation of dietary treatment.

*Diet *At 6 weeks of age, rats were divided into 1 of 4 dietary treatment groups: control (CON); 50% saturated fat (SAT); 40% saturated fat + 10% n-6 PUFA composed primarily of linoleic acid (LA) (SAT+LA) and 40% saturated fat + 10% n-3 PUFA composed primarily of α-linolenic acid (ALA) (SAT+ALA). Diets were supplied by Harlan Teklad (Madison, WI), and are detailed in Tables 1 and 2. The duration of dietary treatment was 32 weeks. Body weight was measured weekly. Rats were fasted overnight prior to terminal sample collection.

**Table 1:** Macronutrient composition and caloric density of diets

	CON	SAT	SAT+LA	SAT+ALA
**Protein % kcal**	20	20	20	20

**Carbohydrate % kcal**	69	28	28	28

**Fat % kcal**	11	52	52	52

**Saturated fat % total FA**	43	93	79	79

**Monounsaturated fat % total FA**	8	4	4	4

**Polyunsaturated fat % total FA**	49	3	16	16

**Kcal/g**	3.6	4.5	4.5	4.5

CON, Control; SAT, 50% saturated fat; SAT+LA, 40% saturated fat + 10% n-6 PUFA; SAT + ALA, 40% saturated fat + 10% n-3 PUFA.

**Table 2:** Fatty acid composition of diets (% of total diet)

	CON	SAT	SAT+LA	SAT+ALA
**8:0**	0.14	2.03	1.80	1.80

**10:0**	0.09	1.35	1.20	1.20

**12:0**	0.72	10.8	9.60	9.60

**14:0**	0.24	3.60	3.20	3.20

**16:0**	0.28	2.51	2.10	1.98

**18:0**	0.24	3.59	2.58	2.58

**18:1 n-9**	0.33	1.01	1.04	0.96

**18:2 (LA)**	1.95	0.85	4.06	3.11

**18:3 (ALA)**	0.00	0.00	0.20	1.38

**n-6**	1.95	0.85	4.06	3.11

**n-3**	0.00	0.00	0.20	1.38

**n-6:n-3**	N/A	N/A	20.3:1	2.26:1

LA, Linoleic acid; ALA, α-linolenic acid; N/A, not applicable; CON, Control; SAT, 50% saturated fat; SAT+LA, 40% saturated fat + 10% n-6 PUFA; SAT + ALA, 40% saturated fat + 10% n-3 PUFA.

### General anesthesia

General anesthesia was induced by placing rats in a commercial rodent anesthesia chamber and initiating flow of 3% isofluorane in a 95% O_2_/5% CO_2 _gas mixture. Anesthesia was maintained by nosecone at 2% isofluorane for noninvasive measurements, and at 4% isofluorane for terminal sample collection.

### Serum measurements

Serum leptin, glucose and free fatty acid (FFA) concentrations were measured in the University of Colorado Hospital Clinical and Translational Research Center. Leptin was measured using the Rat Leptin Radioimmunoassay Kit (Millipore, St. Charles, MO). Glucose and FFA were quantitated using the Roche Cobas Mira Plus Chemistry Analyzer (Indianapolis, IN). Insulin was quantitated by a commercial rat insulin ELISA (Linco Research, St. Charles, MO). The Homeostasis Model Assessment (HOMA) was used to estimate insulin resistance using a HOMA2 IR Excel-based calculator (http://www.dtu.ox.ac.uk/homacalculator/download.php). Serum triglycerides (TG) were assayed using enzymatic colorimetry (Triglycerides Reagent, Thermo Electron, Pittsburg, PA).

### Systolic blood pressure and heart rate

Immediately upon moving to the maintenance dose of 2% isofluorane, rats were moved to a temperature controlled platform for determination of heart rate (HR) and systolic blood pressure by the tail cuff method (SC 1000 Pressure Analysis System, Hatteras Instruments, Cary, NC). Three separate measurements of both HR and systolic blood pressure were recorded.

### Echocardiographic examination

After HR and blood pressure determination, rats were shaved over the ventral thorax and upper abdomen. A Philips HD-11 ultrasound machine with a 12 mHz pediatric sector transducer was used to image the heart in transverse parasternal and 4-chamber views. Two dimensional, M-mode and Doppler imaging was incorporated to measure LV end-diastolic and end-systolic wall and chamber dimensions and isovolumic relaxation time (IVRT), an index of diastolic function. Left ventricular mass was estimated using a formula adapted from Foppa et al:[28]

where LVID_d _= LV internal diameter during diastole, LVW_cr/d _= cranial LV wall thickness during diastole and LVW_ca/d _= caudal LV wall thickness during diastole.

*Dual Energy X-Ray Absorptiometry (DEXA) *Scans were performed on anesthetized rats at the Colorado State University Veterinary Medical Center using a Delphi A densitometer (Hologic, Inc., Bedford, MA).

### Processing of tissue samples

The heart was exposed through a medial sternotomy. Blood was aspirated from the pulmonary arterial trunk and immediately placed into a collection tube. After 2 hours, the blood was centrifuged at 2095 RCF for 15 minutes. After centrifugation, serum was aspirated and stored at -80°C.

Immediately upon withdraw of the blood sample, the heart was excised and placed in ice cold saline, then quickly dabbed for excess fluid prior to recording of heart weight. While in the iced saline, the heart was dissected to isolate the LV, right ventricle and septum, and isolated tissue weights were recorded. Samples of right and left ventricular and septal myocardium were divided and either snap frozen in liquid nitrogen and stored at -80°C or fixed in 4% paraformaldehyde. After 24 hours in paraformaldehyde, tissues were transferred to 70% ethanol, then trimmed and embedded in paraffin. The mass of visceral adipose was estimated by removing and weighing the mesenteric fat.

### Collagen

Masson's trichrome stain was used for collagen detection in paraffin-embedded tissue sections. A slide from each animal was evaluated at 20X for regions of transversely sectioned cells without artifact or large vessels. Four images per slide, comprising identical total areas among slides, were assessed for % total area that was positive for Masson's staining, using NIH Image J software. Hydroxyproline (a primary amino acid in collagen) was quantitated in frozen septal tissue spectrophotometrically using previously described methods[29].

### Lipid analysis

Oil Red O staining was applied to myocardial cryostat sections. Septal TG were extracted and quantitated using a commercial colorimetric Triglyceride Quantification Kit (BioVision Research Products, Mountain View, CA).

Lipids were extracted from frozen septal tissue using the method described by Matyash et al.[30] Briefly, 0.05 gm tissue samples were pulverized into a powder under liquid nitrogen and placed in a glass homogenizer. Mass spectrometry (MS) grade methanol (0.75 mL) and methyl-tert-butyl ether (MTBE, 2.5 mL) were added and the powdered tissue was homogenized briefly on ice. Samples were capped off under nitrogen gas and incubated at room temperature for one hour, then 0.625 mL of MS grade water was added. After vortexing, samples were capped off under nitrogen and incubated at room temperature for an additional 10 minutes, then centrifuged at 1,000 RCF for 10 minutes. The upper (organic) phase was collected and the sample dried under a stream of nitrogen. Samples were frozen at -80°C until processed. Thin layer chromatography (TLC) was then used to separate out the phospholipid fraction using a 20 cm × 20 cm silica gel TLC plate in a 70:30:1 hexane:ethyl ether:acetic acid solution. The band associated with the phospholipid fraction was scraped from the plate and dissolved in 0.5 ml hexane and 0.5 ml 0.5N KOH. Three ml of 14% BF3-methanol was added and each sample was placed on a heat plate at 70 °C for 1.5 hours to obtain methyl esters in preparation for gas chromatography (GC). The GC analysis was performed using an Agilent 6890 Series Gas Chromatographer (Agilent Technologies, Inc., Santa Clara, CA). The column used was an Agilent Technologies DB-225 30 m × 0.250 mm × 0.25 μm, model 122-2232. The initial temperature of the oven was 100 °C with an initial ramp temperature of 10°C/min for 10 minutes, then 2.5°C/min for 4 minutes and held at 210°C for the remaining 15 minutes for a total run time of 29 minutes. The inlet split ratio was 20:1 with the column at constant flow and an initial flow, pressure and velocity at 2.0 ml/min, 23.86 psi and 44 cm/sec, respectively.

### Myocyte cross sectional area

Sections of LV were stained with hematoxylin and eosin, and cross sectional area was measured using NIH Image J software. Fifty transversely sectioned cells with central nuclei from each of 2 slides per rat were evaluated (i.e. 100 cells/rat). In 5/22 rats, only 50 cells/rat met standards for measurement.

#### Statistics

Initial analyses were conducted using Prism 4.0 for Macintosh (Graphpad Software, Inc., San Diego, CA) and SPSS version 19 (IBM, Somers, NY). Bartlett's test for equality of variance and the Kolmogorov-Smirnov test for Gaussian distribution were applied to all datasets. The Kruskal-Wallis nonparametric test and Dunn's post test were used to analyze non-normal data. Pearson's and Spearman's correlations were applied to normal and non-normal data, respectively. Treatment groups were compared using 1-way ANOVA. When the overall ANOVA F-test p-value was < 0.05, the LSD method for pairwise comparisons was used. Because regional wall thickness during systole and diastole were likely to be highly correlated, multivariate analysis of variance (MANOVA) was used to analyze echocardiographic LV wall thickness variables as a set, prior to analysis of the individual wall thickness variables. Computations were performed using the GLM procedure in SAS software (SAS Institute Inc., Cary, NC) version 9.2. Significance was determined by inspecting the 4 multivariate tests provided. Data are expressed as mean +/- SE; statistical significance was set at p < 0.05.

## Results

Data relevant to body morphometry, organ weights, hemodynamics and serum metabolic indices are presented in Table 3. Rats fed diets supplemented with PUFA, whether LA or ALA, had higher body weights than CON rats; further, SAT + ALA rats had higher body weights compared to SAT rats. There were no differences in % body fat by DEXA or in postmortem visceral adipose mass; however, visceral adipose mass was correlated with body weight (r = 0.69, p < 0.0001). Heart weight and LV weight were similar among groups. Treatment did not alter HR or systolic blood pressure. Serum metabolic indices were unchanged by diet; however, leptin was correlated with % body fat (r = 0.86, p < 0.0001) and visceral adipose mass (r = 0.78, p < 0.0001).

**Table 3:** Data summary including body morphometry, tissue masses, hemodynamics and serum metabolic indices

	CON	SAT	SAT+LA	SAT+ALA	p
**Body morphometry and tissue mass**					

Body wt (gm)	550/11.6	571/12.1	616/12.4*	625/26.7**^#^	0.024

% body fat (DEXA)	20.1/2.58	26.8/2.20	25.5/2.80	26.4/1.78	0.199

Visceral adipose wt (gm)	4.37/.701	6.03/.769	7.23/1.35	7.21/.811	0.145

Visceral adipose:body wt	.008/.001	.011/.001	.012/.002	.011/.001	0.248

Heart wt:body wt	.003/.0002	.003/8.869 e-005	.003/6.234 e-005	.003/4.871 e-005	0.365

Heart wt:brain wt	.804/.037	.798/.022	.833/.018	.845/.033	0.593

LV wt:body wt	.0017/.0001	.0015/6.684 e-005	.0014/3.233 e-005	.0014/8.451 e-005	0.102

**Heart rate and blood pressure**					

HR (bpm)	358/7.66	357/11.0	390/10.4	350/6.91	0.090

SPB (mm Hg)	141/9.08	163/9.81	157/11.3	146/3.87	0.272

**Serum measurements and HOMA**					

Leptin (ng/mL)	4.55/1.34	6.71/1.20	7.42/1.96	7.28/0.811	0.438

Insulin (pmol/L)	93.3/11.1	61.5/9.83	82.6/9.09	79.0/9.32	0.259

Glucose (mg/dl)	167/13.0	176/12.1	183/15.2	177/8.74	0.740

FFA (uEq/L)	509/56.3	441/45.9	434/32.2	386/13.7	0.188

TG (mmol)	.620/.091	.612/.066	.560/.087	.573/.049	0.893

HOMA	5.61/1.29	4.43/.657	5.08/.503	4.74/.512	0.875

Data listed as mean/SE. CON, Control; SAT, 50% saturated fat; SAT+LA, 40% saturated fat + 10% n-6 PUFA; SAT + ALA, 40% saturated fat + 10% n-3 PUFA. *p < 0.05 compared to control; **p < 0.01 compared to control; #p < 0.05 compared to SAT.

Myocardial fatty acid profiles are presented in Table 4. With the exception of palmitic and oleic acids, the tissue composition generally reflected direct dietary intake or intake of precursors.

**Table 4:** Fatty acid profile of myocardial phospholipid fractions

	CON	SAT	SAT+PUFA6	SAT+PUFA3	p
**16:0**	10.40/.1632	7.67/.1118**	8.13/.1026	8.85/.1426	0.003

**18:0**	20.94/.0486	25.96/.1592**	25.21/.1392	24.47/.1712	0.003

**18:1**	8.10/.3686	7.40/.2512	4.24/.1463*	4.43/.1312	0.009

**18:2**	18.96/.8193	14.25/.3933	18.96/.4124	18.62/.7574	0.035

**18:3**	.1361/.0251	.1269/.0050	.0715/.0026	.4635/.0190^++^	0.010

**20:4**	26.15/.6430	29.85/.3703	24.22/.4919	19.13/.3994^##^	0.004

**22:5**	.4004/.0321	.3316/.0341	1.711/.1507	4.754/.1906^##^	0.004

**22:6**	2.416/.1158	2.910/.0752	8.557/.2385	14.27/1.387**	0.003

**23:1**	6.21/.705	5.50/.699	3.03/.426	0/0**^#^	0.004

Data listed as mean/SE. CON, Control; SAT, 50% saturated fat; SAT+LA, 40% saturated fat + 10% n-6 PUFA; SAT + ALA, 40% saturated fat + 10% n-3 PUFA. *p < 0.05 compared to control; **p < 0.01 compared to control; #p < 0.05 compared to SAT;

## p < 0.01 compared to SAT; ++ p < 0.01 compared to SAT+PUFA6.

Myocardial outcomes are listed in Table 5. Multivariate analysis of cranial wall dimensions during systole and diastole revealed significant differences in cranial wall measurements based on all 4 multivariate tests (p = 0.009-0.038). The 1-way ANOVA tests of systole and diastole separately revealed that cranial LV wall thickness was increased in rats from both PUFA-supplemented groups compared to CON animals. Moreover, rats fed both SAT + LA and SAT + ALA diets had increased cranial wall thickness during diastole compared to rats fed the SAT diet, and rats fed the SAT + LA diet also had increased cranial wall thickness during systole compared to rats fed the SAT diet. Correlations between cranial LV wall thickness measurements and % body fat by DEXA or visceral adipose weight were weak to nonexistent (Table 6). Caudal LV wall measurements during systole and diastole were not different based on MANOVA (p = 0.164-0.372). Left ventricular mass, estimated from echocardiographic data and indexed to body weight, was similar among groups. This estimate of LV mass correlated to body weight and visceral adipose mass, but not to overall adiposity as measured by DEXA (Table 6). Systolic and diastolic functional indices (i.e. fractional shortening and IVRT, respectively) were not different between groups. Dietary treatment did not alter myocardial TG or collagen content. Oil Red O staining was negligible across treatment groups (data not shown). Cardiac myocyte cross sectional area was increased in all fat-fed groups compared to control; however, there was no difference in area between the fat-fed groups. There was no correlation between body weight or visceral adipose mass, and measures of TG, hydroxyproline or myocyte area.

**Table 5:** Summary of echocardiographic measurements, myocardial hydroxyproline and triglyceride content and myocyte area

	CON	SAT	SAT+LA	SAT+ALA	p
**Echocardiographic measurements**					

LVW_cr/s _(cm)	.309/.008	.329/.009	.371/.018**^#^	.355/.012*	0.013

LVW_cr/d _(cm)	.203/.005	.201/.003	.230/.010*^#^	.227/.008*^#^	0.021

LVW_ca/s _(cm)	.289/.013	.315/.016	.327/.016	.300/.010	0.272

LVW_ca/d _(cm)	.185/.012	.198/.008	.201/.013	.206/.011	0.633

LVID_d _(cm)	.824/.027	.799/.009	.798/.018	.809/.030	0.868

LVID_s _(cm)	.501/.026	.456/.018	.418/.034	.473/.028	0.234

LV mass:body wt	.0029/.0001	.0028/8.950 e-005	.0028/7.855 e-005	.0028/8.966 e-005	0.695

IVRT (sec)	.026/.001	.029/ < .001	.027/ < .001	.027/ < .001	0.265

FS (%)	39.3/1.54	42.9/2.00	47.8/3.47	41.7/1.91	0.112

**Myocardial collagen and triglyceride; myocyte area**					

Hydroxyproline (ug/mg dry wt) n = 4-6	.753/.261	.555/.112	.638/.128	.661/.117	0.837

Triglyceride (nMol/gm wet wt) n = 3-6	9.47/3.67	12.4/1.88	9.11/2.12	9.28/1.21	0.676

Collagen (%) n = 5-6	.055/.004	.053/.005	.045/.003	.044/.004	0.254

Myocyte cross sectional area (microns^2^) n = 5-6	492/30.5	628/23.2**	602/25.1*	610/33.8*	< 0.018

Data listed as mean/SE. LVW, left ventricular wall; LVID, left ventricular internal diameter; IVRT, isovolumic relaxation time; FS, fractional shortening; cr, cranial; ca, caudal; s, systole; d, diastole; CON, Control; SAT, 50% saturated fat; SAT+LA, 40% saturated fat + 10% n-6 PUFA; SAT + ALA, 40% saturated fat + 10% n-3 PUFA.

*p < 0.05 compared to control; **p < 0.01 compared to control; #p < 0.05 compared to SAT.

**Table 6:** Summary of correlation data comparing key myocardial outcomes to adiposity and body weight

Correlation	r	p
LVW_cr/s _and % body fat (DEXA)	0.23	0.309

LVW_cr/s _and visceral adipose wt	0.42	0.058

LVW_cr/s _and body wt	0.47	0.031

LVW_cr/d _and % body fat (DEXA)	0.08	0.722

LVW_cr/d _and visceral adipose wt	0.47	0.034

LVW_cr/d _and body wt	0.45	0.042

LV mass and % body fat (DEXA)	0.21	0.361

LV mass and visceral adipose wt	0.51	0.017

LV mass and body wt	0.67	< 0.001

LVW, left ventricular wall; cr, cranial; ca, caudal; s, systole; d, diastole.

## Conclusions

The aim of this study was to develop insights into the heterogeneity of OC. Accordingly, we sought to identify a profile of related gross and cellular myocardial processes that may be specific to dietary fatty acid composition, using dietary obese SD rats. This study revealed diet-specific changes in myocardial fatty acid composition, LV thickening and myocyte hypertrophy without associated changes in myocardial function.

### Morphometric, hemodynamic and metabolic profiles

In the present study, only the PUFA-fed rats had increased body weight compared to control animals, and a difference associated with dietary fat composition was demonstrated in that SAT + ALA-fed rats had greater body weights than rats fed the SAT diet alone. Increased visceral adipose mass (or "visceral adiposity"), compared to increased body weight, is a stronger risk factor for the development of LVH, OC and failure[31-33]. Both the fatty acid composition[34] and mass[32,35] of the visceral adipose determine the potential for this depot to secrete factors that are believed to contribute to OC[32,36-38]. The present study revealed only a trend toward increased body fat and visceral adipose mass in fat-fed rats. Though differences in primary myocardial outcomes must be interpreted in the absence of significant treatment differences in adipose mass, it is possible that the secretory profile of the visceral adipose was altered according to dietary influence on fatty acid composition and gene expression[39-41].

In the present study, dietary fatty acid composition did not modify systolic blood pressure or HR, suggesting that primary outcomes may be interpreted in the absence of increased afterload. Similarly, serum glucose, serum insulin and calculated HOMA were unchanged by dietary treatment. Though fasting concentrations and subsequent HOMA calculations offer only gross approximations of insulin sensitivity, these findings indicate that neither hyperglycemia nor hyperinsulinemia are likely to be key factors influencing primary outcomes.

### Myocardial fatty acid composition

Dietary fatty acids determine the fatty acid composition of the myocardium, and changes in the type of myocellular lipids are associated with altered intracellular signaling, including pathways that may be important in modulating myocyte metabolism, hypertrophy, contractile function and ultimately survival[42-46]. Overall, comparisons of tissue fatty acid profiles between studies must be made cautiously due to variance in diets and lipid fractions studied. Further, the complex interplay of dietary fatty acids and rates of uptake, oxidation and metabolism is beyond the scope of this paper. With these limitations in mind, the fatty acid composition of the total phospholipid fraction in CON rats was similar to that described in another study of SD rats,[47] with the exception of less myocardial DHA in this study, likely attributable to differences in dietary content. The data support the idea that stearic acid (18:0) is more readily incorporated into the myocardial phospholipid fraction compared to palmitic (16:0) and oleic (18:1) acids,[48] and that increased available LA (18:2) may be preferentially incorporated, resulting in displacement of oleic and palmitic acids. The observed increase in myocardial DHA[49,50] and decrease in arachidonic acid (AA 20:4) [49-51] with ALA feeding have been reported previously.

### Myocardial structure and function

Effects of dietary fats on myocardial structure and function in the setting of pressure overload have been demonstrated[52-54]. Less is known about the role of dietary fatty acid composition in OC, without concomitant hypertension, myocardial ischemia or diabetes. In the present study, LV thickening was associated with PUFA feeding (SAT + LA and SAT + ALA groups) in the absence of hyperglycemia, hyperinsulinemia or hypertension. The effect of high-fat diet composition was demonstrated in that PUFA-fed rats had greater thickening than rats fed the SAT diet. The LV thickening in PUFA-fed rats was regional; the cranial (anterior) wall, but not the caudal (posterior) wall, was affected. These data, combined with poor or nonexistent correlation of cranial LV thickness with adiposity and body weight, suggest that diet may be more important than morphometry in the development of focal LV thickening. Heart and LV masses were not different among treatment groups, suggesting either focal areas of thickening that did not contribute remarkably to overall mass in PUFA-fed rats, or replacement of normal parenchyma with a matrix of lesser density. Unchanged myocardial TG and hydroxyproline content (discussed below) supports the former idea. Correlative data suggest that in contrast to measures of focal LV thickening, total LV mass may be better predicted by visceral adipose mass and body weight than by diet. The LV thickening present in PUFA-fed rats was not associated with *in vivo *systolic or diastolic dysfunction measured echocardiographically. These findings are consistent with those in human studies describing increased LV mass without concomitant dysfunction in obese individuals[55]. Comparing these data with those from other rodent studies, increased heart weight and impaired function, as measured in isolated papillary muscles and myocytes, were reported in rats in response to short-term high-fat feeding[56,57]. In contrast, *in vivo *studies using echocardiography have revealed no change in myocardial structure and function in response to 8 weeks of high SAT and PUFA feeding,[58] but others observed increased LV mass and impaired contractile function in mice fed a high fat diet for 20 weeks[59]. It is likely that chronicity, distribution and underlying etiology of LVH combine to determine subsequent function.

Though it is not uncommon for LVH to exist in the absence of measureable functional change, some factors should be considered relevant to the present study. It is possible that more sensitive echocardiographic indicators of myocardial function, such as tissue Doppler imaging and related methods,[10] may reveal early and subtle functional changes attributed to dietary obesity that are not measureable with conventional echocardiographic techniques used in the majority of studies to date. Additionally, it is possible that despite normal systolic and diastolic function at rest, conditions of increased workload or myocardial stress would reveal impaired function[56]. Regarding dietary treatment chosen for this study, it is possible that any beneficial effects of n-3 supplementation were obscured by concomitant feeding of high saturated fat[60]. It should also be considered that dietary simple carbohydrates, rather than fatty acid composition, play a prominent role in promoting the cardiomyopathic phenotype. Short term effects of a high-fat, high-simple carbohydrate diet were demonstrated in dietary obese Wistar rats that developed myocardial hypertrophy and impaired systolic and diastolic function with just 16 weeks of dietary treatment[61,62]. Finally, it is acknowledged that a limitation of this study that precludes definitive correlation of myocardial composition with function was the use of interventricular septal tissue for measurement of myocardial fatty acids, hydroxyproline and TG, given that gross structure and function were measured in the cranial and caudal LV free walls. Regional differences in myocardial protein expression,[63] substrate uptake[64] and hypertrophy[10] have been demonstrated. It is therefore not valid to assume that changes in the septum wholly reflect those observed in the LV free wall.

With these considerations in mind, it would be erroneous to conclude that diet-induced changes in myocardial fatty acid composition are unassociated with functional impairment. It is widely appreciated that dietary n-3 PUFA are protective against cardiomyopathy,[65,66] and that diets enriched in n-6 PUFA are associated with exacerbation of processes relevant to cardiomyopathy and heart failure[24,67]. It is likely that changes in oxidative stress and inflammation, as well as aberrant myocyte metabolism, were present but not manifest as resting dysfunction detectable echocardiographically.

### Myocyte cross sectional area

Regarding the potential contributors to LVH, namely myocyte hypertrophy, ECM remodeling and lipid accumulation, this study showed that myocyte cross sectional area was increased with feeding of all high-fat diets, regardless of composition. These observations are consistent with those of obese humans. Right heart endocardial biopsies obtained from markedly obese patients with heart failure, mostly attributed to dilative cardiomyopathy, revealed that the most common histologic lesion was mild myocyte hypertrophy that was not described as causative, present in 67% of obese subjects[68]. Evidence of myocyte hypertrophy was also the predominant finding in hearts of obese individuals without premortem evidence of heart disease[12]. This, along with our finding that myocyte hypertrophy did not accompany LVH in SAT rats, suggests that while myocyte hypertrophy is the most consistently identified myocardial lesion in obese individuals, its presence is not likely to solely contribute to clinically relevant LVH. Hypertrophic stimuli, and subsequent genotypic and phenotypic responses, are very diverse[69,70]. Certainly a measure of cross sectional area only defines the presence of the phenomenon, and it is likely that myocyte gene expression, signaling pathways and subsequent preservation or deterioration of structure and function are different according to fatty acid milieu,[71] degree of adiposity, adipokine profile[72] and metabolic aberrancy.

### Myocardial extracellular matrix remodeling

In addition to myocyte hypertrophy, this study investigated ECM remodeling and lipid accumulation as potential contributors to LVH. ECM remodeling is present in failing hearts regardless of etiology,[73] and it is well documented that altered ECM composition contributes to myocardial pathology. Fibrosis, however, is not uniformly present in hypertrophic hearts of obese individuals[12]. In the present study, long-term high fat feeding was not associated with increased myocardial collagen. These data are consistent with short-term studies that measured unchanged interstitial collagen in response to moderate- and high-fat feeding[74,75]. In contrast, other studies revealed that Wistar rats fed either a high-fat or high-fat, high-simple carbohydrate diet for ≈16 weeks had increased myocardial collagen staining[61,62,76]. In addition to diet composition, serum leptin concentrations may impact myocardial ECM homeostasis. In cultured cardiac myocytes, leptin increased collagen expression and matrix metalloproteinase activity[77]. The absence of hyperleptinemia in the rats of the present study may lend partial explanation for ECM preservation.

### Myocardial lipid accumulation

There is disparate evidence regarding the occurrence and relevance of TG (i.e. neutral lipid) accumulation in obesity. Evidence suggests that overweight and obese individuals have increased myocardial TG deposition compared to lean subjects[78,79]. In contrast, LV tissue from humans with end-stage nonischemic heart failure revealed no difference in intramyocardial lipid staining in hearts from lean and obese subjects[79]. Further, postmortem examination of 12 obese individuals without evidence of hypertension or myocardial ischemia identified only scant fatty infiltration in 3 of the subjects[12]. Regarding functional relevance, greater myocardial lipid has been linked with LVH and systolic dysfunction,[78,80] while reduced myocardial lipid was associated with attenuated apoptosis and fibrosis[81]. In contrast, other work suggests that TG accumulation may be protective when alternative pathways lead to formation of harmful bioactive products. Study of cultured cells showed that incubation with oleic acid drives accumulation as TG and preserves cell viability, while exposure to palmitic acid leads to ceramide accumulation and apoptosis[82]. Rodent high-fat feeding studies reveal both increased [56,83] and unchanged myocardial TG[84]. Findings of the present study are consistent with the latter, and expand the observation to include high-fat diets of variable fatty acid composition. Given the trend toward increased TG content in rats fed the SAT diet, however, additional work is warranted to determine whether this observation may represent a diet-specific effect.

### Strain and model considerations

When possible, the above discussion has focused on models of OC without concomitant genetic anomalies or induced pathology (i.e. aortic banding, spontaneous hypertension). Within these studies, the data collectively suggest that there may be strain differences and variability in whole animal vs. *ex vivo *outcomes. Regarding strain, Wistar and SD rats have distinct lipid metabolism,[85] and Wistars may develop myocardial pathology with shorter dietary interventions[56,86]. Additional strain differences in metabolic and myocardial responses to high-fat feeding have been demonstrated[87,88]. Regarding model type, evidence suggests that *ex vivo *studies of OC reveal more profound pathology than observed *in vivo*, and this has been partly attributed to endogenous protective mechanisms[21]. While contractile dysfunction may have been present in isolated muscles or cells from the rats in this study, overall gross systolic and diastolic function was seemingly intact. As noted, there may be distinct signaling pathways that are specific to predominant dietary fatty acids but result in overtly similar outcomes. For this reason, it will be important to subsequently characterize genotype and major hypertrophic pathways to investigate potential differences at the cellular level.

### Limitations

Study limitations include the aforementioned use of interventricular septal tissue for measurement of myocardial fatty acids, hydroxyproline and TG, given that echocardiographic measurements of structure and function were derived from images of the cranial and caudal LV free walls. Additionally, it is likely that increasing sample size and dividing subjects into obesity-prone and obesity-resistant groups would have reduced variability within treatment groups and more clearly elucidated significant differences. Further, echocardiographic assessment under conditions of myocardial stress (i.e. dobutamine administration) may have revealed dysfunction that was not detectable under resting conditions used in the present study. Finally, our understanding of the temporal effects of diet on outcomes would have been improved by conducting measurements over time. By doing this, more accurate comparisons with short-term studies could be made, and potential homeostatic/compensatory mechanisms engaged with long-term feeding could be identified.

### Conclusions

The findings of this study suggest that, under conditions of high-fat feeding, replacement of 10% saturated fat with either LA or ALA is associated with increased body weight and segmental LV wall thickness in the absence of myocardial functional changes. Increased myocyte size, similar among all fat-fed groups, appears to be a more likely precursor to measureable LV thickening in uncomplicated dietary obesity than collagen accumulation or lipid accretion; however, increased myocyte size did not determine gross LV hypertrophy. Predicted responses to PUFA type were not actualized in the outcomes measured in the present study; thus, future studies will measure myocardial gene and protein expression in response to diet, to determine whether hypertrophic pathways are differentially regulated and possibly predictive of a physiologic versus pathologic LV response. Inclusion of simple carbohydrates as part of a western diet in rodents should be further investigated as a relevant model of diet-induced OC in humans, specifically in relation to LVH as a precursor to functional decline.

## Competing interests

The authors declare that they have no competing interests.

## Authors' contributions

KMJ critically reviewed the manuscript for intellectual content, developed the hydroxyproline assay method, and assisted with data analysis. KEM assisted in terminal sample collection, processed tissue and serum samples and performed triglyceride assays. AJC participated in echocardiographic data acquisition and interpretation, and critically reviewed the manuscript for intellectual content. PLC was the principal contributor to statistical design and analysis. CMM performed TLC-GC. PHF measured myocyte cross sectional area. MLM assisted in hydroxyproline assay development and completion. MJP critically reviewed the manuscript for intellectual content. MF conceived of and designed the study, performed echocardiographic examinations and terminal sample collection, and drafted the manuscript. All authors have read and approved the final manuscript.
